# Susceptibility and transcriptional characteristics of inbred C57BL/6J and outbred ICR mice in CDAHFD-induced MASH models

**DOI:** 10.1186/s42826-026-00272-y

**Published:** 2026-04-08

**Authors:** Jing Zhou, Bingyi Li, Ziyi Guo, Bing Zhou, Yan Lu, Lian Zhu, Jin Lu, Jian Wang, E. Wang, Yao Li

**Affiliations:** 1 Department of Laboratory Animal Science, Shanghai Jiao Tong University School of Medicine, 280 South Chongqing Road, Shanghai, 200025 China; 2 Department of Endocrinology and Metabolism, Minhang Hospital, Fudan University, Shanghai, China; 3 Institute of Metabolism and Regenerative Medicine, Shanghai Sixth People’s Hospital to Shanghai Jiao Tong University School of Medicine, Shanghai, China

**Keywords:** MASH, CDAHFD, Mouse strain, Inbred, Outbred

## Abstract

**Background:**

Metabolic dysfunction-associated steatohepatitis (MASH) is a progressive fibrotic liver disease, the underlying mechanisms of which have not been fully elucidated. Most MASH research relies on diet-induced models, particularly inbred mouse strains such as C57BL/6J. Although inbred strains are commonly used, outbred mice more accurately reflect the genetic diversity of human populations. It was reported that ICR mice were resistant to developing MASH after high-fat diet feeding. However, the effects of choline-deficient, L-amino acid-defined, high-fat diet (CDAHFD) on ICR mice remain unexplored. The CDAHFD is a widely adopted diet-induced model for MASH. In this study, C57BL/6J and ICR mice were fed either a normal diet (ND) or a CDAHFD for 10 weeks. Metabolism-related phenotypes and liver histology assessments were conducted to establish MASH models. RNA transcriptome sequencing of liver samples was performed to identify differentially expressed genes, which were then aligned to the human MASH transcriptome.

**Results:**

We successfully established MASH models via CDAHFD in both C57BL/6J and ICR mouse strains. ICR mice presented transcriptional profiles comparable to those of the C57BL/6J strain and effectively replicated the inflammatory and fibrotic features observed in patients with MASH.

**Conclusions:**

This study revealed that ICR mice are as suitable as inbred C57BL/6J mice for CDAHFD-induced MASH models.

**Supplementary Information:**

The online version contains supplementary material available at 10.1186/s42826-026-00272-y.

## Background

Metabolic dysfunction-associated steatohepatitis (MASH), previously known as nonalcoholic steatohepatitis (NASH), is a severe form of metabolic dysfunction-associated steatotic liver disease (MASLD), which is the new nomenclature for nonalcoholic fatty liver disease (NAFLD) [1]. MASH is characterized by inflammation and fibrosis in addition to steatosis, significantly increasing the risk of progression to end-stage liver diseases such as cirrhosis and hepatocellular carcinoma [2]. While MASLD alone does not increase all-cause mortality [3], it is associated with a remarkable increase in death due to liver-related complications (e.g., cirrhosis and liver failure) and extrahepatic comorbidities (e.g., cardiovascular diseases and various malignancies) [4–6].

The global prevalence of MASLD among adults is estimated to reach 30%, with MASH affecting approximately 3% to 5% of this population [7]. By 2030, the prevalence of MASH and its associated mortality are expected to double [8]. Despite this alarming trend, there are significant challenges in treating MASH, primarily due to the lack of pharmacological therapies, in addition to the recent FDA-approved Rezdiffra, a thyroid hormone receptor-beta agonist [9, 10]. Therefore, preclinical mouse models are urgently needed to improve our understanding of MASH pathogenesis.

The choline-deficient, L-amino acid-defined, high-fat diet (CDAHFD) is a widely used diet-induced model of MASH and subsequent hepatocellular carcinoma (HCC) in lean mice [11, 12]. Compared with the Western diet (WD) and choline-deficient high-fat diet (CDHFD), the CDAHFD results in MASH in a more rapid and aggressive way without inducing obesity or metabolic syndrome. Furthermore, unlike the classical methionine-choline-deficient diet(MCD), the CDAHFD does not lead to significant weight loss, which allows prolonged feeding studies. In C57BL/6J mice, CDAHFD significantly elevated the serum levels of ALT and AST and induced hepatic steatosis, ballooning, inflammation, and fibrosis after 6–12 weeks of feeding [12]. Nevertheless, CDAHFD-fed mice remained insulin sensitive and displayed normal glucose tolerance compared with chow-fed controls [13]. Thus, the CDAHFD model provides a rapid and convenient way to assess MASH, particularly in the search for important signaling pathways associated with the ‘nonobese’ MASH subtype through gene expression profiling.

Different mouse stocks or strains may show varying susceptibilities and phenotypes in response to MASH-inducing diets, through which we can identify superior models or even discover novel molecular mechanisms [14]. MASH studies are typically conducted using inbred mouse strains, such as C57BL/6J, to minimize genetic variability and reduce the number of animals. However, findings using inbred mice may lead to overestimation when extrapolating results to humans because they fail to consider interindividual variability. In contrast, outbred stocks, such as the widely used ICR mice, may better reflect the genetic diversity of human populations [15]. Unlike C57BL/6J mice, ICR mice are resistant to developing hepatic steatosis in response to high-fat diet feeding [16]. Therefore, we sought to determine whether ICR mice exhibit similar resistance to CDAHFD-induced MASH.

In this study, we examined CDAHFD treatment in both C57BL/6J and ICR mice to assess differences in their susceptibility to a MASH-like phenotype and to explore their transcriptional resemblance to human MASH.

## Methods

### Animal experiments and sample collection

Eight-week-old male C57BL/6J and ICR mice were purchased from Lin Chang Laboratory Animal Care in Shanghai and housed in the Department of Laboratory Animal Science, Shanghai Jiao Tong University School of Medicine, China. The animals were maintained under controlled conditions of 21 ± 1 °C, 55 ± 10% humidity, and a 12-hour light/dark cycle. The choline-deficient, L-amino acid-defined, high-fat diet (CDAHFD) was purchased from Research Diets (A06071302; 60% kcal fat, nontrans fats, 0.1% methionine, no added choline, and L-amino acid-defined). C57BL/6J and ICR mice were fed a normal diet (ND) or CDAHFD for 10 weeks. During the study, parameters such as body weight, food intake, fasting blood glucose levels, and random blood glucose levels were measured. The mice were euthanized via isoflurane asphyxiation inhalation and cervical dislocation. Liver tissues were either stored in liquid nitrogen or fixed in paraformaldehyde for histopathological analysis.

### Liver triacylglycerol assay and biochemical analysis

The plasma concentrations of triglycerides (TGs), alanine aminotransferase (ALT), and aspartate aminotransferase (AST) were assessed via commercial assay kits (Kehua Bio-Engineering, Shanghai, China) according to the manufacturer’s guidelines. For quantification of liver triglycerides, approximately 100 mg of liver tissue was homogenized in 1 ml of 5% NP40 solution. The homogenized mixture was then incubated in a water bath at 95 °C for 2 to 5 min and centrifuged at 15,000×g for 15 min. The supernatant was subsequently collected for the determination of TG levels, and the hepatic TG content was normalized to the initial tissue mass. Plasma ALT and AST levels were determined by the UV-lactate dehydrogenase and UV-malate dehydrogenase method respectively.

### Assessment of liver histology

For the light microscopic analysis of liver histology, paraffin-embedded liver tissues were sectioned into thin slices, followed by standard hematoxylin and eosin (H&E) staining. Hepatic fibrosis was assessed by Masson’s trichrome staining using a commercial kit (Servicebio, Shanghai, China, Cat# G1006). For Sirius Red staining, the sections were subjected to a 10-minute hematoxylin treatment, followed by a 60-minute incubation in Sirius Red solution. For Oil Red O staining, the frozen sections were stained with Oil Red O working solution (Servicebio, Shanghai, China, Cat# G1015) for 10 min, washed, and counterstained with hematoxylin for 20 s. Immunohistochemical analysis was performed to assess macrophage activation. The sections were incubated with primary antibodies targeting F4/80 (Cell Signaling Technology, MA, USA, Cat# 70076T; diluted 1:100) for 2 h at room temperature, followed by three washes with PBS. The sections were subsequently treated with horseradish peroxidase-conjugated secondary antibodies (Servicebio, Shanghai, China, Cat# GB23303, diluted 1:200) for 1 h at room temperature.

Liver injury severity was semi-quantitatively assessed using the NAFLD activity score (NAS). This score was derived from the unweighted sum of three histological components: steatosis (0–3), lobular inflammation (0–3), and hepatocyte ballooning (0–2), resulting in a total possible score ranging from 0 to 8.

### RNA extraction and qPCR analysis

Total RNA was isolated from liver tissue via the use of TRIzol reagent (Invitrogen, CA, USA, Cat#15596018CN) according to the manufacturer’s protocol. The extracted total RNA was reverse transcribed to cDNA using PrimeScript™ RT Master Mix (Takara, Japan, Cat#RR036A). Quantitative PCR (qPCR) analysis was performed via a quantitative real-time PCR system (Roche, LightCycler 480) with SYBR Green Master Mix (Takara, Japan, Cat#RR820A). Gene expression levels were normalized to the expression of the 36B4 gene, and cycle threshold (Ct) values were computed via the 2ΔΔ-Ct method. The primers used are presented in Supplementary Table 1.

### RNA-seq and data analysis

The RNA-seq data of the mice generated in this study were deposited in the Gene Expression Omnibus (GEO) database under the accession code GSE278017. RNA sequencing services were provided by Cloudseq Biotech, Inc. (Shanghai, China). RNA libraries were prepared from rRNA-depleted RNA samples using a TruSeq Stranded Total RNA Library Prep Kit (Illumina, San Diego, CA, USA). Library quality was assessed via the BioAnalyzer 2100 system (Agilent Technologies, Inc., USA). Paired-end reads (150 bp) were obtained by sequencing. Data preprocessing, including quality control (Q30) and adapter trimming, was performed via fastp software (v0.23.4). High-quality reads were aligned to the reference genome (UCSC MM10) with HISAT2 software (v2.2.1). Gene level raw counts were then obtained as mRNA expression profiles via featureCount software (v2.0.6). Differential expression analysis between the two groups was conducted via edgeR software (v4.0.16), with a p value < 0.05 and |fold change| ≥ 2 serving as the thresholds for identifying differentially expressed genes (DEGs). Kyoto Encyclopedia of Genes and Genomes (KEGG) analyses were performed on the identified DEGs via the online tool KOBAS [17]. All heatmaps were generated via TBtools-II software [18].

### Statistical analysis

Two-tailed Student’s t tests were used to assess significant differences within each strain (ND vs. CDAHFD). Two-way ANOVA followed by Tukey’s post- hoc test was used to analyze the strain × diet interaction. All the data are presented as the means ± SEMs and were analyzed via GraphPad 8.0. Differences were considered statistically significant at *P* < 0.05.

## Results

### General body parameters of C57BL/6J and ICR mice fed the CDAHFD

**Fig. 1:**
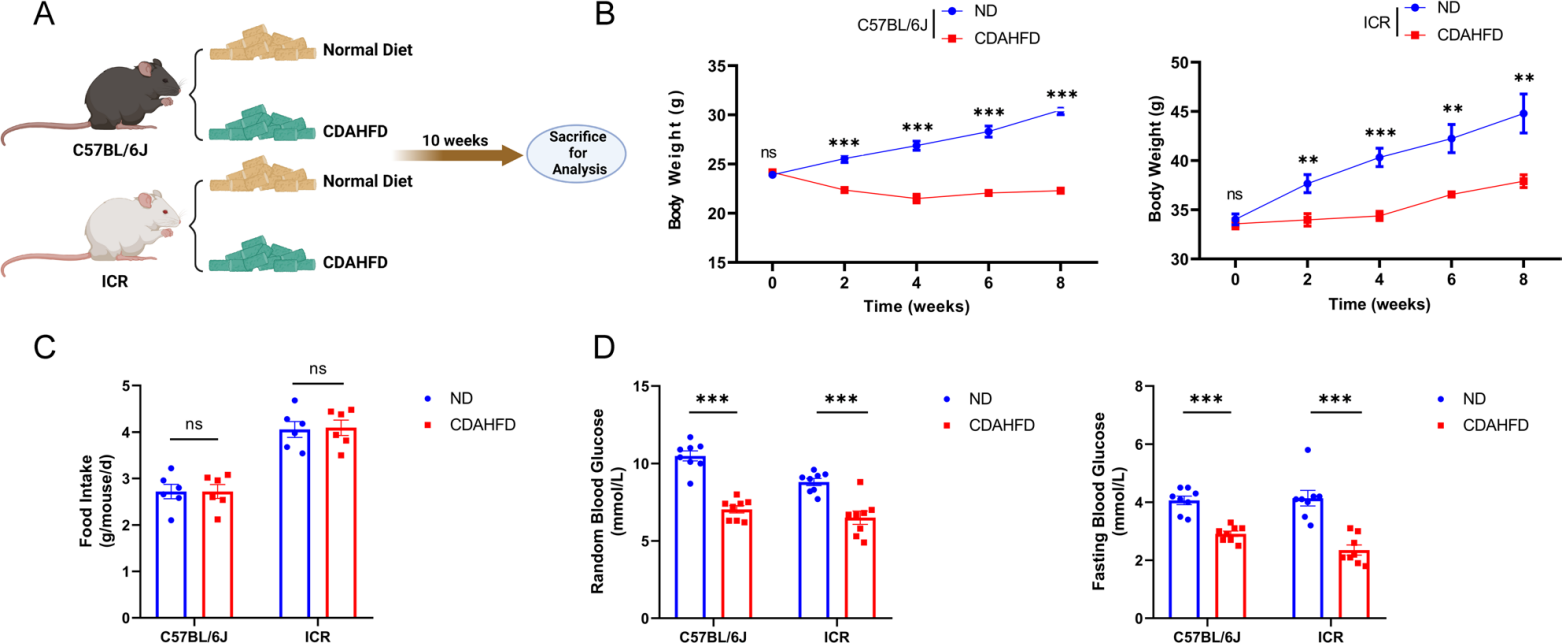
General body parameters of C57BL/6J and ICR mice after CDAHFD feeding. (**A**) Workflow of CDAHFD-induced MASH models of the two mouse strains. (**B**) Body weight, (**C**) food intake, and (**D**) glucose levels of C57BL/6J and ICR mice fed a ND or CDAHFD. The data are presented as the means ± SEMs. *P < 0.05, **P < 0.01, ***P < 0.001, compared with the control group. n = 6–8 per group

To assess the susceptibility of different mouse strains to MASH induced by CDAHFD, male C57BL/6J and ICR mice were fed either a CDAHFD or a normal chow diet (ND) for 10 weeks. Body weight was monitored biweekly, and at the end of the feeding period, all the mice from the four groups were euthanized for subsequent analysis (Fig. 1A).

Compared with their respective ND-fed controls, both C57BL/6J and ICR mice experienced initial weight loss immediately after the CDAHFD was started, which continued throughout the study. Notably, differences emerged between the two strains: C57BL/6J CDAHFD-fed mice presented an 8% reduction in body weight relative to baseline, whereas ICR CDAHFD-fed mice presented a 13% increase in body weight from baseline to the 8_th_ week (Fig. 1B). Both C57BL/6J and ICR mice presented no significant differences in food intake between the two dietary groups (Fig. 1C). In line with previous findings that C57BL/6J mice fed a CDAHFD are highly sensitive to insulin and exhibit lower blood glucose levels [13], our results revealed a decrease in blood glucose levels in both CDAHFD-fed C57BL/6J and ICR mice compared with their respective control groups (Fig. 1D).

### Liver markers and metabolic serum parameters in response to CDAHFD exposure

**Fig. 2:**
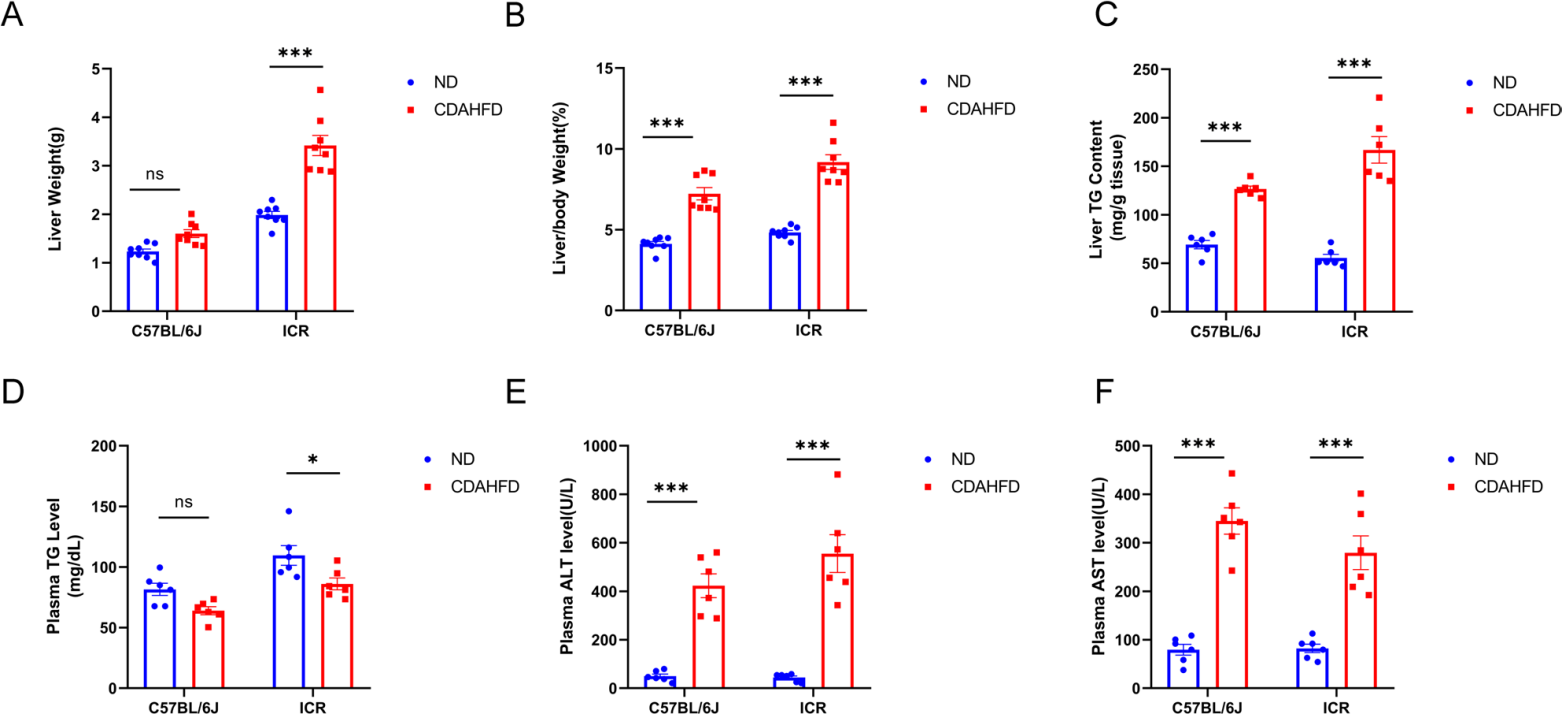
Liver markers and metabolic serum parameters upon CDAHFD exposure. (**A**) Liver weight, (**B**) liver-to-body weight ratio, (**C**) hepatic TG content, and (**D**-**F**) plasma TG, ALT, and AST levels in the 4 groups of mice. *P < 0.05, **P < 0.01, ***P < 0.001, compared with the control group. n = 6–8 per group

The liver weight and liver-to-body weight ratios were significantly increased in CDAHFD-fed C57BL/6J and ICR mice at the end of week 10 (Fig. 2A-B). Compared with those in the respective ND groups, the total liver triglyceride (TG) levels were markedly elevated in the CDAHFD-fed mice, indicating lipid accumulation (Fig. 2C). Moreover, CDAHFD-fed ICR mice presented slight reductions in serum TG levels following CDAHFD administration (Fig. 2D). C57BL/6J mice fed the CDAHFD presented a substantial increase in liver injury biomarkers, with ALT levels reaching 420 ± 50 IU/l and AST levels reaching 340 ± 25 IU/l. Similarly, ICR mice fed the CDAHFD presented increased ALT (550 ± 80 IU/l) and AST (280 ± 35 IU/l) levels compared with those in the untreated control group (Fig. 2E-F).

Two-way ANOVA revealed that the serum TG, ALT, and AST levels were influenced by the main effect of diet, with no significant strain × diet interaction observed. In contrast, a significant interaction was detected for the liver TG content. Post-hoc analysis confirmed that although the two mouse strains showed comparable liver TG levels on a normal diet (ND), ICR mice exhibited a significantly greater increase in hepatic TG following CDAHFD feeding than C57BL/6 mice.

### Induction of hepatic steatosis, inflammation and fibrosis by CDAHFD feeding

**Fig. 3:**
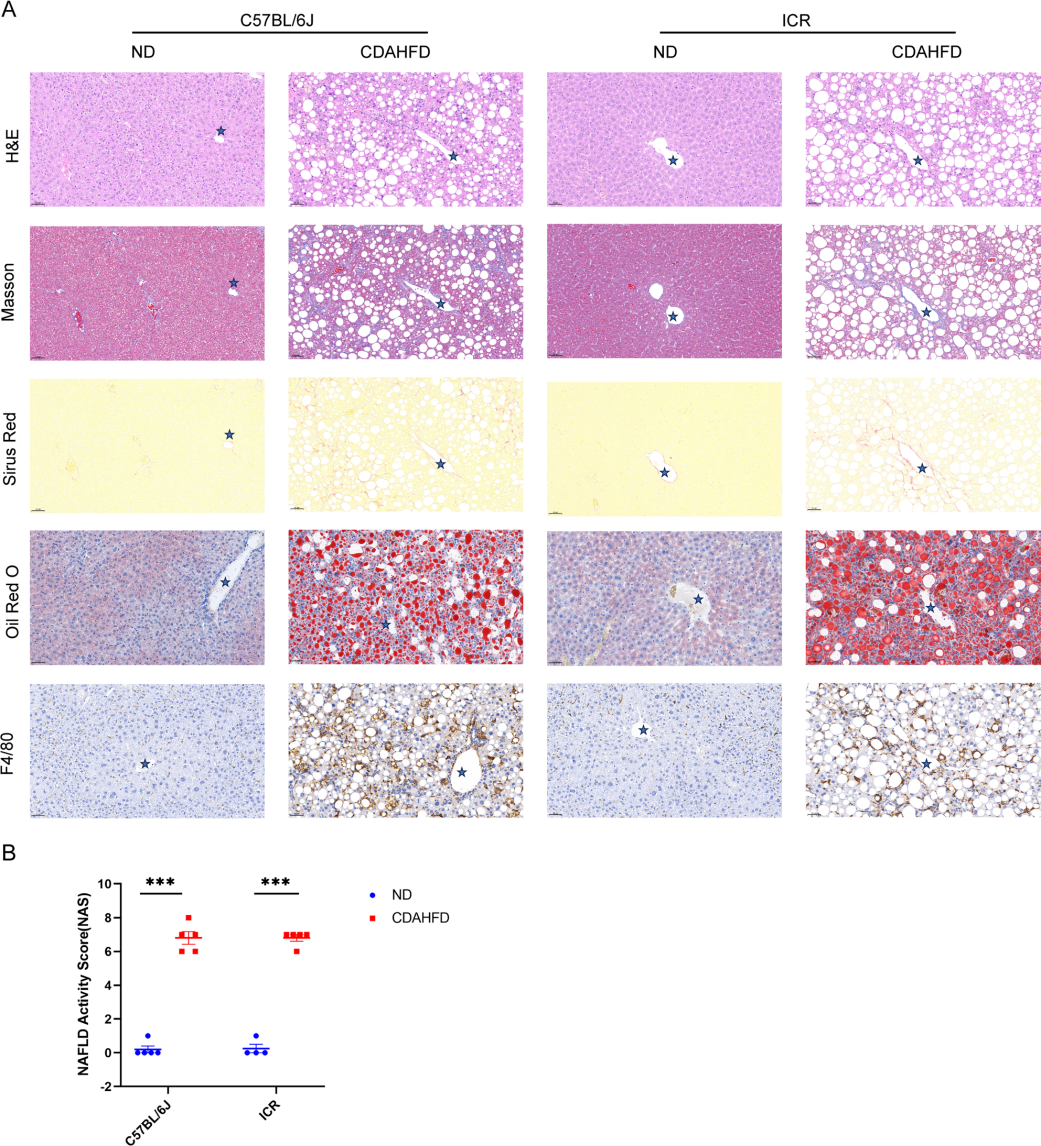
Representative histopathology and immunohistochemistry results for the liver. (**A**) H&E staining, Masson staining, Sirius Red staining, Oil Red O staining, and F4/80 immunohistochemistry of liver sections from the 4 groups of mice. The scale bar represents 50 μm. The stars represent central veins. (**B**) NAS of liver sections from the 4 groups of mice

Liver H&E and Oil Red O staining further confirmed hepatic steatosis and ballooning in CDAHFD-fed C57BL/6J and ICR mice, which was consistent with strongly increased TG accumulation within the liver. However, there was no significant difference in the severity of ballooning observed between the two types of CDAHFD-fed mice. Inflammatory clusters consisting of markedly accumulated Kupffer cells and hypertrophied macrophages were visualized via F4/80 immunohistochemistry. The number of F4/80-positive macrophages was apparently comparable between the two groups of CDAHFD-fed mice, indicating similar levels of infiltration and activation of Kupffer cells. Masson’s trichrome and Sirius Red staining demonstrated that CDAHFD resulted in severe hepatic fibrosis in both C57BL/6J and ICR mice (Fig. 3A).

We further calculated the NAFLD activity score (NAS) to assess the severity of liver injury (Fig. 3B). There was no significant difference in NAS between the two types of CDAHFD-fed mice. Therefore, our data demonstrated that we successfully established MASH models in both types of mice, and they did not show significant differences in liver histology.

### Identification of differentially expressed genes in the two MASH models via RNA-seq data

**Fig. 4:**
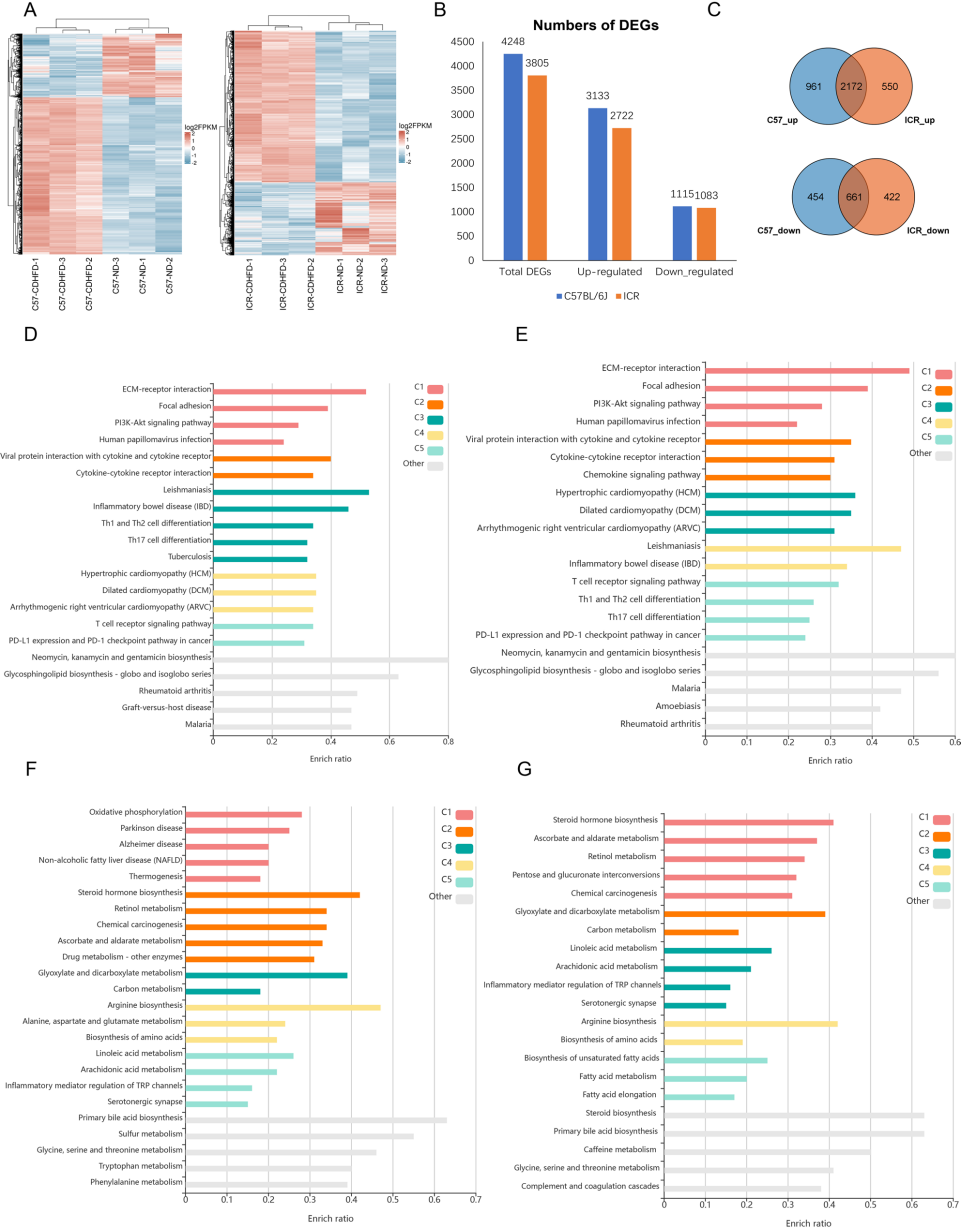
Transcriptome analysis of liver tissues from CDAHFD-fed and ND-fed mice. (**A**) Heatmap showing the expression patterns between different models after batch effect correction. (**B**) The distribution of upregulated and downregulated DEGs detected in each model. (**C**) Common upregulated and downregulated genes detected in the C57BL/6J and ICR groups. (**D**) KEGG analysis of upregulated DEGs in the CDAHFD vs. ND groups of C57BL/6J mice. (**E**) KEGG analysis of upregulated DEGs in the CDAHFD vs. ND groups of ICR mice. (**F**) KEGG analysis of downregulated DEGs in the CDAHFD vs. ND groups of C57BL/6J mice. (**G**) KEGG analysis of downregulated DEGs in the CDAHFD vs. ND groups of ICR mice

Because C57BL/6J and ICR mice presented similar body parameters, serum biochemical profiles, and histological characteristics after CDAHFD feeding, we further conducted RNA sequencing on liver tissues to determine whether they share similar molecular characteristics in terms of transcription. Hierarchical clustering analysis and heatmap representations revealed distinct gene expression patterns, with samples within each group demonstrating close clustering (Fig. 4A). For the identification of differentially expressed genes (DEGs), we employed a threshold of a fold change ≥ 2 and a p value < 0.05. In C57BL/6 mice fed the CDAHFD, we identified 3133 upregulated genes and 1115 downregulated genes compared with those in the control group. In the CDAHFD-fed ICR strain, a total of 3805 DEGs were identified, comprising 2722 significantly upregulated genes and 1083 downregulated genes (Fig. 4B). All the DEGs are listed in supplementary Table 2.

We subsequently conducted a comprehensive analysis of the commonly differentially expressed genes across the two models. A total of 2172 upregulated DEGs (58.97%) and 661 downregulated DEGs (43.00%) were shared between the two mouse types (Fig. 4C), indicating that they had analogous transcriptomic profiles. Kyoto Encyclopedia of Genes and Genomes (KEGG) analysis was performed on the DEGs induced by CDAHFD in each group, revealing the shared involvement of numerous biological pathways (Fig. 4D-G). The two strains shared 90% of the top pathways among the upregulated DEGs and 80% among the downregulated DEGs. The most significantly upregulated genes in both strains were predominantly associated with fibrosis, encompassing pathways such as the ECM-receptor interaction, focal adhesion, and PI3K-Akt signaling pathways. DEGs related to carbon metabolism, steroid hormone biosynthesis and fatty acid metabolism were notably downregulated in the livers of the MASH models. These results indicate that the CDAHFD-induced MASH models share similar molecular mechanisms, despite the differences in mouse types.

### The upregulated DEGs in the two MASH models are closely related to the human MASH transcriptome

Because the transcriptomes of hominine MASH are characterized by the upregulation of genes involved in fibrosis and inflammation, we collated publicly accessible human NAFLD/MASLD transcriptomes and identified upregulated DEGs from a dataset comprising 304 patients [19], aligned these human genes to the corresponding mouse genes via Ensembl, and used them as benchmarks. The upregulated DEGs were categorized in the early stages of MASH (that is, mild vs. control), during the progression of MASH (moderate-severe vs. mild) and throughout all stages of the disease (across all comparisons within the two datasets). All the upregulated DEGs are listed in Supplementary Table 3.

**Fig. 5:**
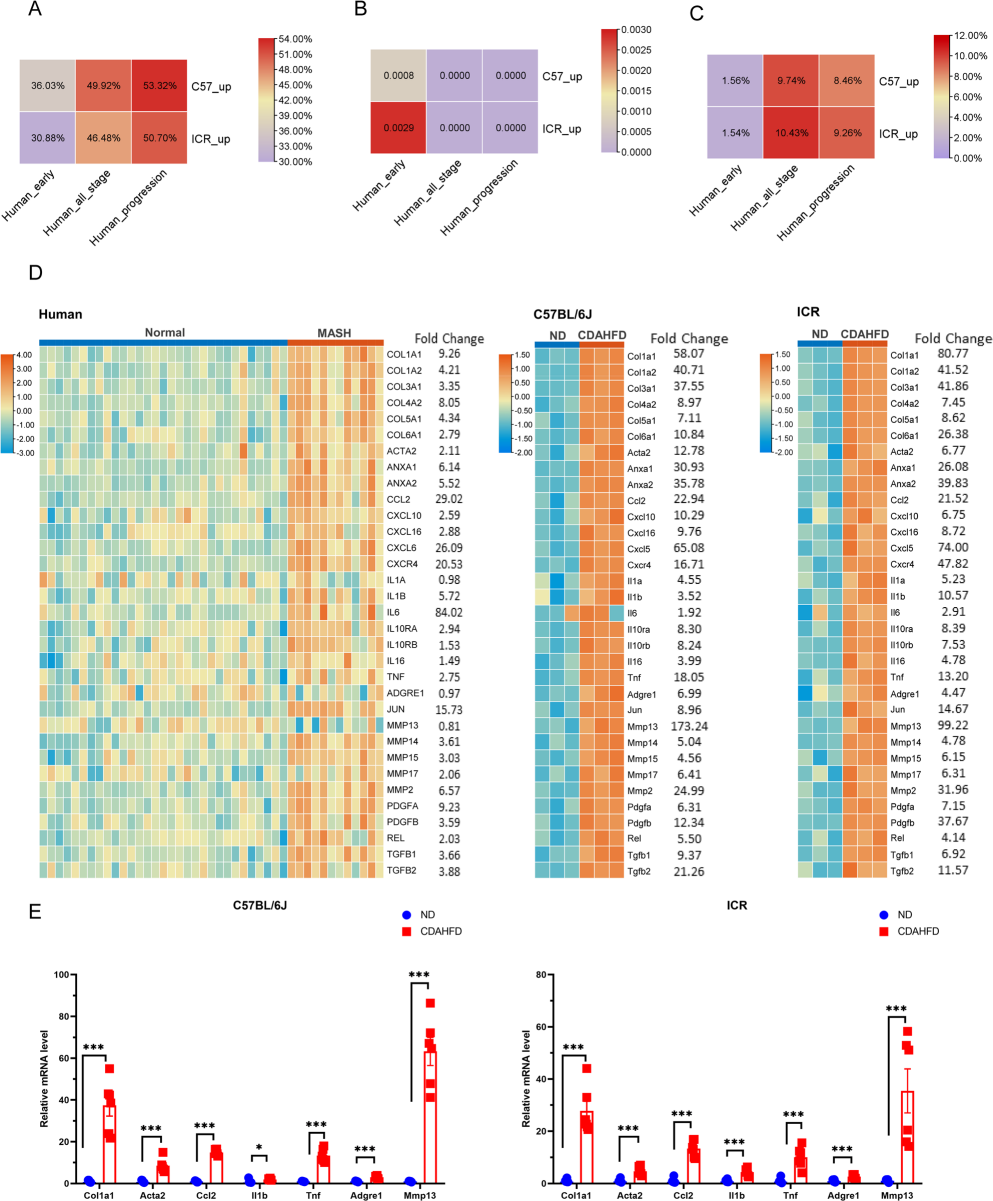
Agreement of mouse DEGs with human MASH. (**A**) Heatmap showing the agreement between mouse MASH models and human data based on significant DEGs. (**B**) The results of the hypergeometric test (one-sided) performed on the comparison groups. (**C**) The proportions of upregulated human DEGs among the upregulated mouse DEGs. (**D**) Heatmap illustration of the expression profiles of genes involved in inflammation and fibrosis in human, C57BL/6J and ICR mice. (**E**) Relative mRNA levels of genes were validated by qPCR

The two CDAHFD models were compared to human MASH transcriptomic data to evaluate their alignment in terms of upregulated DEGs. The alignment rate is defined as the proportion of human DEGs that are also identified as DEGs in our mouse model. The comparison of both models demonstrated a high level of agreement with the human data. The alignment rates of ‘early disease development’, ‘all disease stages’ and ‘disease progression’ of human upregulated DEGs gradually improved from approximately 30% to nearly 50% in both mouse types, indicating that CDAHFD models more accurately represent ‘disease progression’ or ‘the late stage’ of MASH than ‘the early stage’ (Fig. 5A). Furthermore, the p values derived from the one-sided hypergeometric test applied to the comparison groups demonstrated that the upregulated DEGs of both the C57BL/6J and ICR mouse models exhibited statistically significant proximity to the hominine MASH transcriptomes (Fig. 5B). We subsequently calculated the proportions of human upregulated homologous DEGs among all upregulated murine DEGs and performed a chi-square analysis (Fig. 5C). Compared with those in C57BL/6J mice, the proportions of homologous DEGs associated with ‘all disease stages’ and ‘disease progression’ in ICR mice were marginally elevated (10.43% vs. 9.74% and 9.26% vs. 8.46%, respectively). Although no statistically significant differences were detected between the two mouse strains (P values were 0.3995 and 0.3033, respectively), the ICR models may exhibit a higher detection rate for DEGs associated with human MASH. In contrast to the C57BL/6J strain, the outbred ICR stock is more similar to humans in terms of genetic heterogeneity, which is characterized by a large gene pool and a high heterozygosity rate. Consequently, the total number of identified DEGs in ICR mice was lower than that in C57BL/6J mice in our study (Fig. 4B). This may have contributed to a higher detection rate by reducing the incidence of false positives.

We tested whether the inflammatory and fibrotic gene expression patterns of the livers of the CDAHFD model mice were similar to those of the livers of MASH patients. We compared the expression levels of these genes in MASH patients with stage 4 fibrosis with those with normal liver histology using data from GSE162694 (Fig. 5D). The majority of these genes had the same direction of regulation (upregulated) and larger fold changes relative to human subjects, indicating that the CDAHFD models in both mouse strains effectively replicated the inflammatory and fibrotic features observed in patients with MASH. All of the above inflammatory and fibrotic genes were validated by qPCR analyses (Supplementary Table 4). The corresponding changes in the levels of inflammatory cytokines (*IL1b*, *Ccl2*, and *Tnf*) and fibrosis-related markers (*Col1a1*, *Acta2* and *Mmp13*) are showed in Fig. 5E.

Consequently, CDAHFD models of ICR mice may serve as useful tools for studying the molecular pathogenesis of MASH.

## Discussion

There are numerous genetically induced, diet-induced, and toxin-induced preclinical models of MASH, with diet-induced models being the most widely used. Although not all of these models faithfully phenocopy and mirror human pathology, diverse diet-induced models are needed for a better comprehension of the pathogenesis of MASH.

MASH is frequently associated with obesity [2, 20, 21], but it is increasingly acknowledged that certain individuals develop MASH in the absence of obesity, especially in Asia [22, 23]. Here, we analyzed ‘nonobese’ MASH in mice fed a choline-deficient, methionine‐lowered, L‐amino acid‐defined high‐fat diet (CDAHFD).

The models for ‘nonobese’ MASH have been modified over time. Traditionally, methionine- and choline-deficient diets (MCDs) have been widely used in ‘nonobese’ MASH models. MCD-induced MASH is characterized by rapid and severe progression of fibrosis, accompanied by significant systemic weight loss [24]. In contrast, the CDAHFD was developed as a modified diet of MASH and has been used and favored by many investors [12, 13, 25–27]. Recent studies have indicated that the CDAHFD reduces the hepatic levels of phosphatidylcholines (PCs) and acylcarnitines (ACs), which is supported by metabolic analysis and is in line with the tendency of human MASH [28].

The strain of mice utilized can have a profound effect on disease outcome. Numerous studies have documented variations in susceptibility to diet-induced obesity and MASH among different murine strains. Even substrains of C57BL/6J mice may differ in their tendencies toward obesity, insulin resistance, MASH and subsequent liver cancer development [29, 30]. However, findings in this area have sometimes been ambiguous or contradictory. For example, A/J mice displayed a more severe MASH phenotype when fed an MCD diet than did C57BL/6J mice [31]. Conversely, when fed a CDAHFD, A/J mice exhibited a slower progression of MASH, with reduced inflammatory cell infiltration and fibrosis than C57BL/6J mice [12]. In a previous study, ICR mice were found to be resistant to developing hepatic steatosis in response to high-fat diet feeding, unlike C57BL/6J mice [16]; however, in our study, ICR mice demonstrated comparable susceptibility to CDAHFD-induced MASH compared with C57BL/6J mice. This discrepancy may stem from the different responses of ICR mice to various MASH-inducing diets.

## Conclusions

We successfully established MASH models through the administration of the CDAHFD to C57BL/6J and ICR mice within 10 weeks. The ICR mice exhibited comparable susceptibility, transcriptional profiles and detection rates of DEGs associated with human MASH to those of the C57BL/6J strain. Given that ICR mice display a more representative variability as seen in the human population, we propose that the CDAHFD-induced ICR mouse is a promising MASH model, particularly for the ‘nonobese’ subtype.

## Supplementary Information

Below is the link to the electronic supplementary material.

Supplementary Material 1: Supplementary Table 1: The primer sequences.

Supplementary Material 2: Supplementary Table 2: All the DEGs of CDAHFD-fed C57BL/6J and ICR Mice.

Supplementary Material 3: Supplementary Table 3: Upregulated DEGs in MASH patients.

Supplementary Material 4: Supplementary Table 4: The values of 2ΔΔ-Ct of representative genes validated by qPCR.

## Data Availability

Further information and requests for resources and reagents should be directed to and will be fulfilled by the lead contact Yao Li (yao.li@shsmu.edu.cn).

## Abbreviations

MASH: Metabolic dysfunction-associated steatohepatitis
NASH: Nonalcoholic steatohepatitis
MASLD: Metabolic dysfunction-associated steatotic liver disease
NAFLD: Nonalcoholic fatty liver disease
NAS: NAFLD activity score
CDAHFD: Choline-deficient, L-amino acid-defined, high-fat diet
ND: Normal diet
WD: Western diet
CDHFD: Choline-deficient high-fat diet
MCD: Methionine–choline-deficient diet
ALT: Alanine aminotransferase
AST: Aspartate transaminase
TGs: Triglycerides
DEGs: Differentially expressed genes
KEGG: Kyoto Encyclopedia of Genes and Genomes
